# Late Presentation of HIV Infection: Prevalence, Trends, and the Role of HIV Testing Strategies in Guangzhou, China, 2008–2013

**DOI:** 10.1155/2016/1631878

**Published:** 2016-09-27

**Authors:** Weibin Cheng, Weiming Tang, Zhigang Han, Thitikarn May Tangthanasup, Fei Zhong, Faju Qin, Huifang Xu

**Affiliations:** ^1^Department of AIDS/STD Control and Prevention, Guangzhou Center for Disease Control and Prevention, No. 1, Qide Road, Jiahe, Baiyun District, Guangzhou, Guangdong Province 510440, China; ^2^University of North Carolina Project-China, No. 2, Lujing Road, Yuexiu District, Guangzhou, Guangdong Province 510095, China

## Abstract

*Background*. The prevalence, trends, and the role of different HIV testing strategies in late presentation of HIV infection in China were unknown.* Methods*. Data of newly reported HIV cases in Guangzhou between 2008 and 2013 was analyzed to examine the prevalence, trends, and characteristics of late presentation of HIV infection by three types of HIV testing strategies.* Results*. Overall, 53.2% (1412/2653) and 27.3% (724/2653) met the criteria of late presentation and presentation with advanced HIV disease. The overall trend of late presentation of HIV infection within the study period was declining. Late presentation was 62.9% in 2008 and dropped to 43.3% in 2013 (*P* < 0.001); presentation with advanced HIV disease was 40.3% in 2008 and dropped to 15.2% in 2013 (*P* < 0.001). Of the three testing strategies, PITC presented higher odds of both late presentation [AOR (95% CI): PITC versus VCT: 1.37 (1.09, 1.73); PITC versus MHT: 3.09 (2.16, 4.42)] and presentation with advanced HIV disease [AOR (95% CI): PITC versus VCT: 1.65 (1.29, 2.11); PITC versus MHT: 13.14 (8.47, 20.39)].* Conclusions*. Although the late presentation of HIV infection was declining, it was still high in Guangzhou. The worse situation among PITC cases urges the policy adjustment in medical settings to increase early HIV diagnosis.

## 1. Background 

In October 2009, the European Late Presenter Consensus working group reached a consensus definition of late presentation of HIV infection [1]. According to the consensus, late presentation was defined as persons presenting for care with a CD4 cell count below 350 cells/*μ*L or presenting with an AIDS-defining event, regardless of the CD4 cell count. Presentation with advanced HIV disease was defined as persons presenting for care with a CD4 cell count below 200 cells/*μ*L or presenting with an AIDS-defining event, regardless of the CD4 cell count. Late presentation for HIV care is detrimental for infected persons and others [2–4]. Studies showed that late presentation not only has a serious impact on HIV transmission [5–8], but also has become the major concern for AIDS and AIDS-related deaths in western countries [9, 10]. Despite tremendous efforts, a substantial number of late HIV infections still persist worldwide [11–15]. Even though we already have essential data from the United States and European countries, there is a lack of data on late presentation of HIV infection in China and other Asian countries.

A number of studies documented that late presentation of HIV infection is generally more common among marginalized individuals (i.e., ethnic minorities, drug users, and immigrants), people with low perceived risk of HIV infection (e.g., the elderly and heterosexuals), and those of low socioeconomic status (e.g., lower educational attainment) [14, 16–24]. Late presentation of HIV infection reflected the lack of knowledge about HIV and/or cultural and social stigma and socioeconomic barriers which limited the access to healthcare. While HIV testing strategy is considered to be important for late diagnosis as it offers opportunities for HIV positive identification, there is scarce data about testing strategies.

In the past decade, with the help of international funding agencies, the availability of testing services in China has increased remarkably. In 2004, a free HIV voluntary counseling and testing (VCT) program was introduced to China, which followed the “Four Free, One Care” HIV policy [25, 26]. VCT services are mainly available at the Centers for Disease Control and Prevention (CDC) and primary healthcare centers that are equipped with trained staff. VCT is free of charge, targeting people who have experienced risk of HIV infection. However, people need to take the initiative to drop by the center to use the service. To increase the VCT service uptake, CDC has implemented a series of intervention programs among the at-risk population, such as men who have sex with men (MSM) and female sex workers. In 2005, mandatory HIV testing (MHT) was launched at detention centers, methadone maintenance treatment clinics, and reeducation/labour camps [27]. MHT was administered to all individuals when they entered into the detention settings. The purpose of MHT was to test at-risk groups including drug users, prisoners, and sex workers along with their clients. In 2006, provider-initiated testing and counseling (PITC) was introduced and implemented at general hospitals and STI clinics in China [26]. HIV test was prescribed by the clinical doctors in the hospital when patients presented with HIV clinical indicator diseases. Meanwhile, PITC included applying HIV testing to individuals who presented to STD clinics. PITC was a paid HIV testing service by using an “opt-out” approach. Patients were informed that HIV testing will take place along with other standard tests and that they must state whether they do not wish to be tested. These three HIV testing strategies are the main types of HIV testing strategies in China. However, according to our knowledge, the effectiveness of testing strategy in late HIV diagnosis has not been evaluated.

Given the clinical and public health benefits of early HIV diagnosis on timely antiretroviral therapy (ART) initiation, it is imperative to understand the situation of late presentation of HIV infection in China and to provide evidence on whichever strategies could facilitate early diagnosis of HIV infection. Thus, by analyzing the HIV case report data in Guangzhou in 2008 to 2013 that was extracted from the Chinese HIV/AIDS Comprehensive Response Information Management System (CRIMS) [28], we conducted this study to examine the prevalence and trend of late presentation of HIV infection and to explore the role of different testing strategies in late presentation of HIV infection.

## 2. Methods 

HIV testing service in Guangzhou city is available within authorized CDC, medical institutes, primary healthcare centers, and collective settings. By the end of 2008, there were three HIV confirmatory test laboratories and ninety-one HIV screening laboratories (this increased to 147 in 2013).

Guangzhou started HIV/AIDS case reporting through the Internet in 2005. By the end of 2007, CRIMS was developed and put into use. It is a web-based real-time database system managed by the National Centre for AIDS/STD Control and Prevention (NCAIDS), China CDC [28]. CRIMS includes case report module and epidemiology follow-up module. An electronic record is created in CRIMS for each patient who tested positive for HIV. Case report and epidemiology follow-up services are conducted by trained staffs from hospitals, district level CDC, and primary healthcare centers.

### 2.1. Data Sources

The data included in our study was retrieved from CRIMS case report module and epidemiology follow-up module. The data included information on demographic characteristics, sexual and drug use behaviors, transmission routes, medical histories, and laboratory test results. In this study, we only included cases that were over 15 years old when identified and were identified between January 1, 2008, and December 31, 2013, in our data analysis.

### 2.2. Statistical Analysis

In this study, first-time CD4 cell count results (tested within two weeks after confirmatory HIV positive test) were used to define late presentation of HIV infection. Late presentation was defined as CD4 cell count below 350 cells/*μ*L or presenting with an AIDS-defining event at the first follow-up, regardless of the CD4 cell count. Presentation with advanced HIV disease was defined as CD4 cell count below 200 cells/*μ*L or presenting with an AIDS-defining event at the first follow-up, regardless of the CD4 cell count. Descriptive analyses were used to describe and compare (Chi squared test) demographic characteristics of late presentation of HIV infection cases identified through the three types of testing strategies. Trends of late presentation of HIV infection by three types of testing strategies during the study period were examined. Multivariate logistic regression models were used to compare the proportion of late presentation of HIV infection with different testing strategies. Demographic variables with *P* < 0.20 under Chi squared test analysis were adjusted for multivariate logistic regression models. Adjusted odds ratio (AOR) and 95% confidence intervals (95% CI) were also estimated. Results with a two-sided *P* < 0.05 were considered statistically significant. All statistical analyses were performed with SPSS 18.0 for Windows.

### 2.3. Ethics Statement

This study was based on data from the Chinese government HIV/AIDS CRIMS. All individuals signed a general consent form when they enrolled in CRIMS, and no additional study informed consent was needed for this current study. The study protocol was approved by Guangzhou CDC Ethics Committee. All personal identification information was removed prior to analysis.

## 3. Results

Of the 6,737 cases identified between 1 January 2008 and 31 December 2013, 2,653 (39.4%) cases that had CD4 cell count results at the first epidemiology follow-up were included in this study. Demographic characteristics of the cases identified through PITC were tendency to be older (42.1% aged over 40), being female (26.4%), being married (60.2%), and self-reporting being infected through heterosexual transmission (69.8%). VCT cases had higher educational attainment (38.0% had a college degree or above) and were infected through homosexual contact (65.6%). The majority of MHT cases were infected through injecting drug use (86.1%). Overall, 53.2% (1412/2653) and 27.3% (724/2653) were classified into late presentation and presentation with advanced HIV disease groups. PITC cases had a higher proportion of late presentation (70.9% and 51.7% for VCT and 37.7% for MHT) and presentation with advanced HIV disease (50.0% and 19.9% for VCT and 9.9% for MHT). All characteristics were significantly different among the three groups (*P* < 0.001) (Table 1).

The annual proportions of late presentation from 2008 to 2013 were 62.9% (197/313), 49.6% (121/244), 68.0% (259/680), 54.9% (260/474), 48.8% (336/689), and 43.3% (239/552). Although the overall proportion of late presentation declined during the study period (*P* < 0.001), only the annual rate of late presentation among cases identified through VCT was significantly reduced (from 65.4% to 38.1%). Annual proportions of presentation with advanced HIV disease from 2008 to 2013 were 40.3% (126/313), 18.4% (45/244), 28.9% (1137/680), 24.1% (166/689), and 15.2% (84/552). The overall proportion of presentation with advanced HIV disease was declining (*P* < 0.001), while similar results were found for both VCT and PITC strategies (Table 2).

Two multivariate logistic regression models were developed to compare late presentation of HIV infection within three HIV testing strategies. Models were adjusted for year, age, sex, marital status, educational attainment, route of HIV infection, and place of living. Results showed that PITC has the highest odds of late presentation of HIV infection among the three strategies [late presentation, PITC versus VCT, AOR (95% CI): 1.37 (1.09, 1.73); PITC versus MHT: 3.09 (2.16, 4.42); presentation with advanced HIV disease, PITC versus VCT: 1.65 (1.29, 2.11); PITC versus MHT: 13.14 (8.47, 20.39)]. Among the three strategies, MHT has the lowest odds of late presentation of HIV infection (*P* < 0.01) (Table 3).

## 4. Discussion

In this study, we found that half of the HIV newly diagnosed cases showed late presentation of HIV infection. Among the late presenters, half showed presentation with advanced HIV disease. The epidemic of late presentation of HIV infection in China was high [3], compared to Europe, even though the situation has significantly dropped in the past few years. In addition, this study has compared three types of HIV testing strategies role in the late HIV diagnosis. Results showing a high proportion of late presentation of HIV infection among PITC cases urged the need for improving the capacity of early case detection in medical settings.

Delayed diagnosis of HIV infection may result in late initiation of ART, which subsequently could undermine the effectiveness of clinical outcomes. A recent study demonstrated that deferred initiation of ART at CD4 levels 251 to 350 cells/*μ*L was associated with higher rates of AIDS and mortality, compared to starting ART at the range of 351 to 450 cells/*μ*L (HR: 1.28, 95% CI: 1.04–1.57), and that the adverse effect of deferring treatment increased with the decreasing CD4 cell count threshold [29]. Meanwhile, late presentation of HIV infection reflects the inefficiency of the health providers in response to patients' need for access to HIV services, such as testing and treatment [30]. However, early identification presents an opportunity to encourage safer behavior, which in turn reduces the chance of HIV transmission. The seriousness of late HIV diagnosis in Guangzhou presented a great barrier to achieve the UNAIDS goal of ending AIDS epidemic by 2030.

In this study, robust associations were observed between HIV testing strategies and late presentation of HIV infection. Before that, few reports had explored the association between HIV testing strategies and late HIV diagnosis [18, 31, 32]. Consistent with previous studies, results suggested that PITC did not facilitate more timely diagnosis than targeted HIV counseling. As Duffus WA pointed out, clinical risk-based testing strategy (including PITC), even if implemented successfully in their facility, would still have missed an earlier diagnosis most of the time [33]. PITC acted as an alternative strategy that captured individuals who were unwilling to seek or unaware of seeking HIV testing independently [31, 34]. However, this “opt-out” testing for HIV following diagnosis of an indicator disease was an important strategy for HIV infection case finding [18]. To increase the efficacy of early diagnosis of HIV infection, researchers suggested that adjusting routine provider-initiated HIV testing for at-risk groups in settings such as sexually transmitted infection clinics, drug dependency programs, or antenatal care might be more effective [35].

Interestingly, mandatory HIV testing showed great potential in identifying cases early. In Guangzhou, mandatory testing was well targeted at drug users. The ongoing mandatory HIV testing among drug users increased early diagnosis of HIV infection among this population and significantly contributed to the control of HIV epidemic among intravenous drug users in Guangzhou, as well as across China [36]. This result also concurred with the finding of one study conducted in the Republic of Korea [32]. Furthermore, studies indicated that mandatory HIV testing allows healthcare providers to provide early intervention to the at-risk population and to provide opportunities for testing, substance abuse treatment, and ART, which played an important role in the control of HIV epidemic [37, 38].

China Nation Free Antiretroviral Therapy Program (NFATP) was piloted in 2002 and was implemented nationally in 2003. Free ART criteria were started at CD4 cell count of less than 200 cells/*μ*L and increased to less than 350 cells/*μ*L in 2008 [39]. Although the longitudinal trends of late presentation of HIV infection in this study had shown a declining gesture, persistently high prevalence of late presentation (about 60%) was observed among PITC. One important reason is that high level of HIV related stigma/discrimination and low HIV risk perception among at-risk populations hindered the initiative access to HIV services [40]. PITC was mainly applied for the general population in the medical settings, which presented a gap of demanding for intervention to raise HIV awareness among at-risk individuals in the general population. Another common reason for those presenting late was that patients have often been seen with indicator diseases in the recent past by healthcare professionals without prompt offering of an HIV test [41]. These represent missed opportunities for diagnosis. HIV testing should continue to expand across clinical settings to reduce the number of patients living with undiagnosed HIV infection [42].

The limitations of the current reported study include the following: first, more than half of the cases were excluded due to the lack of CD4 cell count results which may compose an unrepresentative sample. Nonetheless, we compared cases that had CD4 cell count result at first follow-up with those that did not; a minor difference of the proportion of cases that progressed to AIDS at the end of the observation year was found between these two groups (32.2% versus 29.1%). Second, the definition of late presentation of HIV infection used in this study was relatively conservative. The interval between HIV diagnosis and CD4 cell count test within two weeks could underestimate and make comparison difficult. However, evidence suggested that although the percentage of HIV-infected persons with late diagnosis increases with longer cut-offs, the general factors associated with late diagnosis may not vary substantially across the different cut-offs [43]. Third, stigma and social desirability may prevent drug users, sex workers, and homosexual men from reporting the transmission route correctly, and this may lead to information bias. Nonetheless, these three HIV testing strategies have been designed to target different population at risk of HIV infection. Understanding the efficiency of different testing strategies on early case finding would be useful for HIV prevention policy adjustment and resources allocation.

Even with these limitations, this study presented important data that pointed out a high prevalence of late presentation of HIV infection among HIV cases identified in Guangzhou. Furthermore, our data demonstrated that voluntary HIV counseling and testing strategies were more effective in early HIV diagnosis. Recently, the national antiretroviral therapy guideline updated initial ART to treat all patients once diagnosis of HIV infection is confirmed. The early identification of HIV infection cases enables treatment to be initiated at a time when responses are optimal, which in turn reduces the chance of transmission [39]. However, more efforts to better understand the optimal HIV testing practice to facilitate early HIV diagnosis are needed.

## Figures and Tables

**Table 1:** Characteristics and proportion of late presentation of HIV infection among newly diagnosed positives by three types of HIV testing strategies between 2008 and 2013 in Guangzhou, China.

Variable	Total (*N* = 2653)	VCT (*N* = 1275)	PITC (*N* = 832)	MHT (*N* = 546)	*P*
	*n* (%)				
Year					
2008	313 (11.8)	156 (12.2)	101 (12.1)	56 (10.3)	<0.001
2009	244 (9.2)	79 (6.2)	75 (9.0)	90 (16.5)	
2010	381 (14.4)	95 (7.5)	194 (23.3)	92 (16.8)	
2011	474 (17.8)	206 (16.1)	160 (19.2)	108 (19.8)	
2012	689 (26.0)	382 (30.0)	179 (21.5)	128 (23.4)	
2013	552 (20.8)	357 (28.0)	123 (14.9)	72 (13.2)	
Age (years)					
<20	75 (2.8)	41 (3.2)	28 (3.4)	6 (1.1)	<0.001
20~	860 (32.4)	535 (42.0)	175 (21.0)	150 (27.5)	
30~	981 (37.0)	438 (34.4)	279 (33.5)	264 (48.4)	
40~	490 (18.5)	180 (14.1)	207 (24.9)	103 (18.9)	
≥50	247 (9.3)	81 (6.4)	143 (17.2)	23 (4.2)	
Sex					
Male	2264 (85.3)	1131 (88.7)	612 (73.6)	521 (95.4)	<0.001
Female	389 (14.7)	144 (11.3)	220 (26.4)	25 (4.6)	
Marital status					
Single	1355 (51.1)	750 (58.8)	228 (27.4)	377 (69.0)	<0.001
Married	1015 (38.4)	396 (31.1)	501 (60.2)	118 (21.6)	
Divorced or widowed	244 (9.2)	117 (9.2)	91 (10.9)	36 (6.6)	
Unknown	39 (1.5)	12 (0.9)	12 (1.4)	15 (2.7)	
Educational attainment					
Primary school/lower	526 (19.8)	113 (8.9)	165 (19.8)	248 (45.4)	<0.001
Secondary school	1534 (57.8)	677 (53.1)	562 (67.5)	295 (54.0)	
College/above	593 (22.4)	485 (38.0)	105 (12.6)	3 (0.5)	
Route of HIV infection					
Male-to-male sexual contact	917 (34.6)	837 (65.6)	77 (9.3)	3 (0.5)	<0.001
Injecting drug use	654 (24.7)	85 (6.7)	99 (11.9)	470 (86.1)	
Heterosexual contact	967 (36.4)	326 (25.6)	581 (69.8)	60 (11.0)	
Others	115 (4.3)	27 (2.1)	75 (9.0)	13 (2.4)	
Place of living					
Urban	1384 (52.2)	827 (64.9)	316 (38.0)	241 (44.1)	<0.001
Rural	480 (18.1)	205 (17.1)	133 (4.4)	133 (24.2)	
Outside of Guangzhou	789 (29.7)	374 (45.0)	172 (31.5)	172 (31.5)	
Late presentation					
Yes	1412 (53.2)	659 (51.7)	590 (70.9)	206 (37.7)	<0.001
No	1241 (46.8)	616 (48.3)	242 (29.1)	340 (62.3)	
Presentation with advanced HIV disease					
Yes	724 (27.3)	254 (19.9)	416 (50.0)	54 (9.9)	<0.001
No	1929 (72.7)	1021 (80.1)	416 (50.0)	492 (90.1)	

VCT: HIV voluntary counseling and testing; PITC: provider-initiated testing and counseling; MHT: mandatory HIV testing.

**Table 2:** Changes in late presentation of HIV infection over time by three types of testing strategies in Guangzhou, 2008–2013.

	2008 (*N* = 313)	2009 (*N* = 244)	2010 (*N* = 381)	2011 (*N* = 474)	2012 (*N* = 689)	2013 (*N* = 552)	*P*
	Late presentation, *n* (%)						
Total	197 (62.9)	121 (49.6)	259 (68.0)	260 (54.9)	336 (48.8)	239 (43.3)	<0.001
Testing strategy							
VCT	102 (65.4)	37 (46.8)	55 (57.9)	114 (55.3)	172 (45.0)	136 (38.1)	<0.001
PITC	78 (77.2)	43 (57.3)	161 (83.0)	106 (66.3)	120 (67.0)	82 (66.7)	0.052
MHT	17 (30.4)	41 (45.6)	43 (46.7)	40 (37.0)	44 (34.4)	21 (29.2)	0.131

	Presentation with advanced HIV disease, *n* (%)						
Total	126 (40.3)	45 (18.4)	166 (43.6)	137 (28.9)	166 (24.1)	84 (15.2)	<0.001
Testing strategy							
VCT	63 (40.4)	11 (13.9)	26 (27.4)	49 (23.8)	63 (16.5)	42 (11.8)	<0.001
PITC	59 (58.4)	24 (32.0)	126 (64.9)	77 (48.1)	91 (50.8)	39 (31.7)	0.002
MHT	4 (7.1)	10 (11.1)	14 (15.2)	11 (10.2)	12 (9.4)	3 (4.2)	0.294

VCT: HIV voluntary counseling and testing; PITC: provider-initiated testing and counseling; MHT: mandatory HIV testing.

**Table 3:** Comparison of different testing strategies on late presentation of HIV infection by multivariate logistic regression^+^.

	Late presentation AOR (95% CI)	*P*	Presentation with advanced HIV disease AOR (95% CI)	*P*
VCT	Ref.		Ref.	
PITC	1.37 (1.09,1.73)	0.008	1.65 (1.29, 2.11)	<0.001
MHT	0.32 (0.23, 0.46)	<0.001	0.13 (0.08, 0.20)	<0.001

PITC versus MHT	3.09 (2.16, 4.42)	<0.001	13.14 (8.47, 20.39)	<0.001

AOR: adjusted odds ratio; CI: confidence intervals.

VCT: HIV voluntary counseling and testing; PITC: provider-initiated testing and counseling; MHT: mandatory HIV testing.

^+^Models were adjusted for year, age, sex, marital status, educational attainment, route of HIV infection, and place of living.

## Abbreviations

HIV: Human immunodeficiency virus
AIDS: Acquired immune deficiency syndrome
VCT: HIV voluntary counseling and testing
PITC: Provider-initiated testing and counseling
MHC: Mandatory HIV testing
STI: Sex transmitted infection
CDC: Centers for Disease Control and Prevention
ART: Antiretroviral therapy
CRIMS: Chinese HIV/AIDS Comprehensive Response Information Management System
NCAIDS: National Centre for AIDS/STD Control and Prevention
STD: Sex transmitted disease
AOR: Adjusted odds ratio
CI: Confidence intervals
HR: Hazard ratio
NFATP: Nation Free Antiretroviral Therapy Program.
